# Workshop on ethics and communication in Copenhagen 11–13.3.2007

**DOI:** 10.1186/1476-069X-7-S1-S1

**Published:** 2008-06-05

**Authors:** Lisbeth E Knudsen, Domenico Franco Merlo, Ann Dyreborg Larsen

**Affiliations:** 1 Department of Environmental Health, Institute of Public Health, University of Copenhagen, Oester Farimagsgade 5, KD 1014K, Copenhagen, Denmark; 2 Unit of Epidemiology and Biostatistics, National Cancer Institute, Genova, Italy

## Workshop on ethics and communication in Copenhagen 11–13.3.2007

The European Environment and Health Strategy adopted by the European Commission in 2003 presented a new vision on how to address environment and health in an integrated way by putting health in the centre of environmental policy. Based upon the Strategy the Commission adopted in 2004 a Communication on the Environment and Health Action Plan 2004 – 2010. In Action 3 of this Action Plan the European Commission announced to develop a coherent approach to Human Biomonitoring in Europe in close cooperation with the Member States.

The first step (2004–2006) in implementation of Action 3 consisted of the technical preparation of the European Pilot Project. For this reason the EU Commission launched the ESBIO (Expert team to support biomonitoring in Europe) project, scheduled for 2006 and 2007. The project team consisted of nearly all members of the Implementation Group on Human Biomonitoring.

ESBIO aimed to identify and analyse major ethical problems related to individualised human biomonitoring (HBM) as part of the preparatory work of ESBIO of initiating a European human biomonitoring pilot project. The workshop in Copenhagen was part of the deliverables of ESBIO, which is further described in the presentations of Smolders et al. [1] and Thomsen et al [2]. The workshop provided two days of debate on identifying major items for guidelines for ethical issues and communication in human biomonitoring. Almost 50 participants from most European countries and US attended the workshop and represented stakeholders of industry, regulators, academia and animal welfare. This special issue of Environmental Health includes manuscripts from some of the presenters at the workshop while Philippe Grandjean's presentation is published [3] as well as the presentation by Uffe Lind [4].

### Survey on ethics

ESBIO performed a survey on the general ethical procedures related to individualised human biomonitoring through questionnaires for stakeholders within biomonitoring forwarded to ESBIO members for supplementary information. A Danish template was used and inputs were received from Germany, Belgium, United Kingdom, Sweden, France, Luxembourg, Italy, Estonia, Czech Republic, Slovakia, Portugal and Cyprus. The questionnaires revealed common rules and procedures but also some differences related to extent of information, public involvement and feed back. The results from the survey are available on the ESBIO webpage as the report [5].

The legal situation in a number of countries regarding Ethics committees and data protection was extracted from existing data in Privireal (Privacy in Research Ethics and Law) database [6] and tables with information about country specific regulation were developed and sent for approval to key persons in each country of Denmark, Estonia, France, Germany, Ireland, Lithuania, Netherlands, Poland, Portugal, Slovak Republic, Spain, Sweden, United Kingdom. These pages are available on the ESBIO webpage [5] and examples of practices in Poland [7] are provided in this supplement issue of Environmental Health. The supplement issue also include ethics issues experienced in HBM within Portuguese health surveillance [8].

### Principles of research ethics

Research Ethics is based on the Nuremberg Code, which are ten standards to which physicians shall conform when carrying out experiments on human subject. The code was laid down by the war crimes tribunal after 2^nd ^World War [9]. The Helsinki Declaration followed the Nuremberg Code in June 1964 and acts as a guideline for the contracted states of the World Medical Association [10]. In general the Declaration contains guidelines for requirement prior to initiation of any research, while the research is being conducted and after its completion and the publication of the project and its results. When research is carried out on human beings the interest of the society (gain of knowledge) must be weighed in proportion to the interest of the research subject to safeguard his/her integrity and not endanger or risk life or health. The most important feature of the Declaration is the duty of the researcher working with the test subjects; to safeguard the health of the involved parties above all else (article 2 and 3).

### Research Ethics Committees

Article 13 sets down the procedural guidelines for Research Ethics Committees (REC) and states that research projects must always seek ethical approval before initiation of a project.

Most of the European countries have RECs at a national level, examples of which are described in [5] (Sweden, Lithuania, the Netherlands, Germany, The UK, Portugal, Ireland, Denmark, France, Estonia and the Slovak Republic) [6].

The structure of the institutions approving biomedical activity differs yet they for the most part respect the Helsinki Declaration. In Denmark regional committees are placed in each of the five regions and each includes 12–20 members, half of which are lay persons and the rest health personnel. In Germany there are about 150 regional ethics committees, each including 12–20 widely multidisciplinary members. On Cyprus the National Bioethics Committee is the institution which shall approve the biomedical activities. It is an independent body and includes 13 multidisciplinary members, who are appointed directly by the Council of Ministers for 4-year term. To assist with the evaluation of proposals the National Committee has appointed two 9-members Bioethics Evaluation Committees. And in Belgium ethics committees are associated with universities or hospitals, mostly including biomedical professionals, in some committees maybe also one or two philosophers or lay persons.

### Data protection

Data protection and communication of study findings are essential issues concerning HBM as also stated in several of the papers in this supplement issue of Environmental Health [11-14]. In 1995 the Data Protection Directive 95/46/EC was addressed to the Member States of the European Union . The purpose of this Directive is to protect the fundamental rights and freedoms of natural persons and in particular their right to privacy with respect to the processing of personal data. Informed consent is one of the key components in the Directive and the requirement of obtaining consent has been implemented in all of the above mentioned countries. Withdrawal of consent is not always stated in the Data Protection Act (e.g. in the Netherlands, Germany, Portugal and Slovakia [6]), but it must be assumed that withdrawal may take place at any time during biomedical research. Data processing is a detailed area of data protection and all countries have implemented the provision and guidelines from the Data Protection Directive.

### Communication of results

Environmental Health aspects are predominant with HBM and special attention should be devoted to societal issues as discussed in [15], communication of results as discussed by Keune et al [16] in relation to the Flemish biomonitoring program and by Arendt [11] in relation to breastfeeding and measurements of critical levels in the breast milk. The benefit from breastfeeding with essential supplies to the newborn must be weighed towards potential risks. Interpretation of HBM data related to risk has been discussed internationally and by Boogard et al [17].

The"right to know" may challenge as participants in a biomedical research projects always have the right to acquire information and the researchers have a duty to inform the participants about the information necessary. The participant also has the "right NOT to know", which may be difficult to satisfy if much communication is by feed-back to participants.

### Children

The workshop was a continuation of a stakeholder meeting in Brussels organised by the DG environment of EU, described by Sepai et al [18] where stakeholders in human biomonitoring from industry, government, research and NGOs also were asked about opinions on organisation of biomonitoring studies including children:

• Enrolment could either be through direct approaches to parents or advertisements.

• Repeated measurements were fully acceptable by all and the majority accepted all samplings of blood, urine, scalp hair and the questionnaire.

• Study persons should be informed about study results or be given the opportunity to request results. Data should be protected by coding and made available for governments and research after anonymisation.

• Reimbursement of expenses related to participation should be organised while different views were expressed regarding incentives as gifts and payment.

• National differences in regulation should be respected while harmonisation was considered a necessity for future HBM activities.

### Conclusions and recommendations

Conclusions and recommendations from the workshop to be brought forward to the European projects that hopefully will be initiated soon were:

• Protocols need to be developed covering common issues for all participants in collaborative HBM studies, declaring the study aims and specific hypotheses, the study populations and differences related to environmental exposures – background levels, spot exposures, vulnerable populations

The protocol should also describe methods to be used in:

- Selection and recruitment of study persons (special issue with children – directly via family or through schools),

- Sampling (which media and how much) and

- Processing and storage of samples, analysis

- Results and results interpretation

- Information strategy prior, while and after study

Special attention must be given to the informed consent with elements of

▪ Information about study

▪ Procedures (sampling, questionnaire, monitoring, follow-up, data protection)

▪ Sign to participation in separate parts

▪ Agree to store samples and biobanking/future uses

▪ Agree to use samples for purposes of environmental health studies

▪ Agree to share results with other researchers, policy makers

Finally the workshop addressed the common issue of biobanking which implies thorough information of participants, reporting of study results and potential other uses, insurance of data protection but opportunities of data sharing and follow-up to fully exploit study material and to avoid unnecessary repeats of samplings.

## Competing interests

The authors declare that they have no competing interests.
